# Environment, Migratory Tendency, Phylogeny and Basal Metabolic Rate in Birds

**DOI:** 10.1371/journal.pone.0003261

**Published:** 2008-09-23

**Authors:** Walter Jetz, Robert P. Freckleton, Andrew E. McKechnie

**Affiliations:** 1 Division of Biological Sciences, University of California San Diego, La Jolla, California, United States of America; 2 Department of Animal and Plant Sciences, University of Sheffield, Sheffield, United Kingdom; 3 DST/NRF Centre of Excellence at the Percy FitzPatrick Institute, Department of Zoology and Entomology, University of Pretoria, Pretoria, South Africa; Centre National de la Recherche Scientifique, France

## Abstract

Basal metabolic rate (BMR) represents the minimum maintenance energy requirement of an endotherm and has far-reaching consequences for interactions between animals and their environments. Avian BMR exhibits considerable variation that is independent of body mass. Some long-distance migrants have been found to exhibit particularly high BMR, traditionally interpreted as being related to the energetic demands of long-distance migration. Here we use a global dataset to evaluate differences in BMR between migrants and non-migrants, and to examine the effects of environmental variables. The BMR of migrant species is significantly higher than that of non-migrants. Intriguingly, while the elevated BMR of migrants on their breeding grounds may reflect the metabolic machinery required for long-distance movements, an alternative (and statistically stronger) explanation is their occupation of predominantly cold high-latitude breeding areas. Among several environmental predictors, average annual temperature has the strongest effect on BMR, with a 50% reduction associated with a 20°C gradient. The negative effects of temperature variables on BMR hold separately for migrants and non-migrants and are not due their different climatic associations. BMR in migrants shows a much lower degree of phylogenetic inertia. Our findings indicate that migratory tendency need not necessarily be invoked to explain the higher BMR of migrants. A weaker phylogenetic signal observed in migrants supports the notion of strong phenotypic flexibility in this group which facilitates migration-related BMR adjustments that occur above and beyond environmental conditions. In contrast to the findings of previous analyses of mammalian BMR, primary productivity, aridity or precipitation variability do not appear to be important environmental correlates of avian BMR. The strong effects of temperature-related variables and varying phylogenetic effects reiterate the importance of addressing both broad-scale and individual-scale variation for understanding the determinants of BMR.

## Introduction

Understanding the ways in which organisms allocate energy is fundamental for linking behavioral and life-history traits to evolutionary fitness, and for identifying drivers of physiological adaptation. Basal metabolic rate (BMR) characterizes the maintenance energy requirements of individuals and as such is a core descriptor of a species' energy turn-over rate and, ultimately, its energetic niche in the environment [1]–[3]. BMR is the lower limit of the metabolic scope of a normothermic endotherm and represents maintenance energy demands in the absence of thermoregulatory, digestive or activity-related increases in metabolism [1]–[3]. Both the processes underlying the body mass-dependence of BMR [4]–[6] and the determinants of body mass-independent variation in BMR have proved to be of enduring interest to ecological and evolutionary physiologists [e.g. 7], [8]–[12].

Significant geographic variation in maintenance energy requirements in mammals and birds has been noted. In small mammals (<1 kg), BMR varies along a continuum from high BMR in species inhabiting highly seasonal, colder environments with more predictable rainfall at high latitudes to low BMR in warmer, less predictable habitats in the semi-tropics [8], [10], [13]. These correlations between BMR and physical environments in small mammals have been interpreted in a supply-demand adaptive framework, with low BMR in mammals from warm, arid environments viewed as an adaptive trait that minimizes energy requirements during unpredictable bottlenecks in food supply [8], [10]. Higher BMR in species inhabiting colder, more mesic habitats is thought to facilitate high rates of thermoregulatory heat production during rapid heat loss at low environmental temperatures [8], [10]. For birds, Weathers [14] found that BMR generally increases with latitude toward the poles, and recent studies have shown similar intraspecific patterns [12], [15], [16]. Several recent comparative studies of avian BMR have produced evidence that birds inhabiting desert habitats have evolved lower BMR than their mesic counterparts [17], [18]. A recent study comparing multiple environmental predictors found strong evidence for a negative effect of average annual temperature of capture locations on avian BMR, but none for habitat net primary productivity [19]. This is consistent with similar results for avian field metabolic rates rates [20] and suggests that the major environmental correlate of avian BMR is ambient temperature, with elevated BMR associated with cold environments and vice versa. Such temperature-associated variation in BMR could reflect phenotypic plasticity or genotypic adaption [21]–[25].

In mammals, relative mobility has been identified as a major determinant of selection acting on maintenance energy demands (Lovegrove, 2000). Whereas the BMR of small mammals is correlated with temperature and habitat aridity, the absence of similar correlations in large mammals is attributed primarily to the capacity of larger species to avoid localized energetic shortfalls by migrating [8]. Interspecific variation in relative mobility is even more pronounced in birds, since even small species undertake seasonal long-distance movements, escaping adverse local conditions for parts of the year. Mobility or migratory tendency may affect avian BMR in at least three possible ways: i) long-distance movements may pose specific demands on the energetics of migrating species, e.g. in terms of high rates of energy acquisition in preparation for migration [26]–[29], leading to higher BMR compared to non-migrants; ii) only seasonal presence in the breeding region may remove some of the selection pressures on BMR (e.g. survival of cold winters) that affect non-migrants; iii) given environmental effects on BMR, occupation of different environments alone may cause non-migrant – migrant differences in BMR (migrants tend to breed at higher latitudes with colder temperatures). Several authors have noted that long-distance migrant shorebirds have higher BMR than expected on the basis of body mass alone [30]–[32], but a general test of the effect of migratory tendency on avian BMR and the relative role of environmental effects has been lacking.

Recent work has seen an increased appreciation for intraspecific variation in BMR, specifically individual variation due to phenotypic plasticity [33]–[35]. In addition to short-term thermal acclimations, individual birds may adjust BMR as a component of seasonal acclimatization (e.g. to cold winter temperatures) or as part of their migratory cycle [34]. For select migrant species the individual BMR has been shown to vary strongly just before and after the breeding season with significant changes during migration [27], [36]. The variation in BMR found across species thus reflects a number of sources of phenotypic variation, including body mass, genotypic adaptation, phenotypic plasticity – and their interactions with climatic conditions - and phylogenetic inertia. To date a common phylogenetic structure has been assumed for the BMR of both migrant and non-migrants, but migratory tendency may cause different relative strengths of a phylogenetic signal due to different degrees of within-individual variation. In non-migrants little seasonal variation in BMR is expected before winter [34], but in migrants BMR on breeding grounds may vary strongly within and between individuals in the context of their migration. We hypothesize that this variation may affect the strength of the correlates of interspecific BMR variation and potentially lead to a weaker phylogenetic signal in migrants compared to non-migrants.

In this study, we use an extensive global dataset to analyze the influence of migratory tendency on the magnitude and environmental correlates of avian BMR. Specifically, we ask the following questions: i) Across a broad set of species, do migrants generally have higher BMR than non-migrants? ii) What are the strongest environmental predictors of BMR? iii) Can a potential effect of migratory tendency on BMR alternatively be explained by their occupation of different environments? iv) Do migrants and non-migrants show intrinsic differences in their BMR-environment relationships? Finally - and over-arching all of these issues - v) what is the role of phylogeny in shaping these relationships and is it stronger in non-migrants than migrants?

## Results

**Figure 1:**
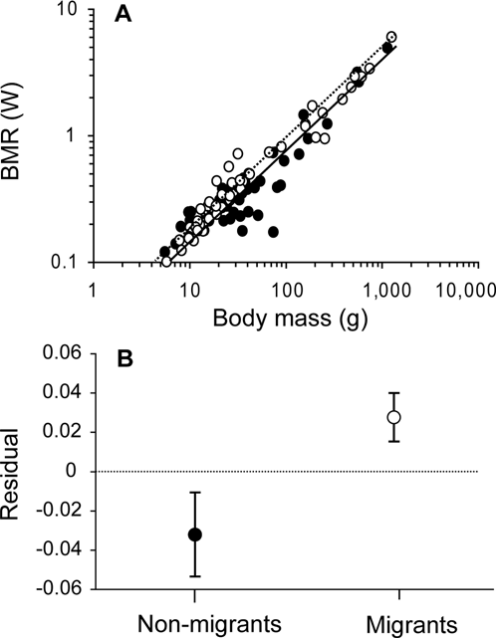
Avian BMR increases with body mass, and is higher in migrants than non-migrants. A Individual data points and partial regression fits for non-migrants (black, solid line) and migrants (open, dotted line). B average residuals (±s.e.) from the overall regression of log BMR on log body mass for non-migrants and migrants. Full dataset (N = 135).

We first use the full dataset (N = 135) without phylogenetic control to illustrate core correlates of avian basal metabolic rate (BMR) and to demonstrate the significance of migratory tendency. Confirming previous studies we find that in a two-predictor model BMR increases consistently and strongly with body mass (*M*: b = 0.744, t = 30.89, p<0.001) and is additionally significantly higher in Passerines than Non-Passerines (*Pass/non-p*.: b = 0.082, t = 5.595, p<0.001). We first test for a potential effect of migratory tendency on BMR only controlling for body mass (Fig. 1). We find that in migrants BMR is much higher than in non-migrant birds (Migratory: b = 0.044, t = 3.93, p<0.001). The effect of migratory tendency is confirmed in the three-predictor model controlling for *Pass/non-p.* membership which yields the best overall fit (Migratory: b = 0.032, *t* = 3.05, p<0.01, full model adjusted r^2^ = 0.912). Refinement of the binary migratory tendency variable to a three-level distinction of non-migrant, short and long-distance migrants did not significantly improve the model fit suggesting that BMR did not differ between those two broad categories of migration distance.

These effects retain their strength when the dataset is restricted to those populations for which available environmental data allows further analysis (N = 97): not accounting for phylogeny, but controlling for *M* and *Pass/non-p.* migrants have higher BMR than non-migrants (*b* = 0.061, *t* = 2.55, *p* = 0.012). For this dataset we examine the additional effect of phylogeny. We find that when phylogeny is controlled for the migrant/non-migrant difference appears no longer to be significant (*b* = 0.036, *p* = 0.174), with the maximum likelihood value of *λ* being significantly different from zero (*λ* = 0.82; test versus *λ* = 0: *P*<0.001).

We then evaluated whether environment can explain variation in BMR above and beyond these three predictors, specifically within migrants and non-migrants. All of the ten putative environmental correlates we tested are highly correlated with latitude and many also with each other (Table 1). Variables with strongest co-linearity include average annual temperature (*Temp avg*, r≥0.5 for 8 out of 9 relationships) and average net primary productivity (*NPP avg*, r≥0.5 for 5 out of 9 relationships). We identified six which had strong associations with BMR: *NPP avg*, *NPP max*, *Prec avg*, *Temp avg*, *Temp max*, *PET*, and *Temp range* (Table 2). None of these associations became non-significant when phylogeny was controlled for and, indeed, the estimated effects of several were even stronger when phylogeny was controlled for (including, *Temp avg*, *Temp max, PET and Temp range*; Table 2), revealing the complexity of the influence of phylogeny on BMR in this dataset.

**Table 1:** Correlation matrix of environmental variables and species traits in the analysis.

	*M*	*NPP avg*	*NPP max*	*Prec avg*	*Temp avg*	*Temp max*	*PET*	*Aridity*	*Temp range*	*Prec range*	*Prec CV*	*BMR*
*Abs. latitude*	*0.41*	***−0.50***	***−0.50***	***−0.61***	***−0.87***	***−0.65***	***−0.90***	*0.22*	***0.71***	***−0.61***	*−0.09*	***0.51***
*M*		−0.43	−0.43	−0.35	−0.39	−0.36	−0.42	0.09	0.12	−0.34	0.05	**0.95**
*NPP avg*			**0.99**	**0.85**	**0.50**	0.37	0.48	0.48	−0.36	**0.64**	**−0.63**	−0.44
*NPP max*				**0.85**	**0.50**	0.38	0.48	0.48	−0.35	**0.64**	**−0.63**	−0.45
*Prec avg*					**0.52**	0.32	**0.55**	**0.55**	**−0.55**	**0.69**	**−0.57**	−0.39
*Temp avg*						**0.82**	**0.83**	−0.22	**−0.63**	**0.55**	0.15	**−0.51**
*Temp max*							**0.70**	−0.37	−0.15	0.45	0.18	−0.44
*PET*								−0.33	**−0.54**	0.47	0.04	**−0.51**
*Aridity*									−0.16	0.28	**−0.73**	0.14
*Temp range*										−0.48	0.01	0.24
*Prec range*											−0.30	−0.35
*Prec CV*												0.02
												

Abbreviations: BMR – log_10_ basal metabolic rate; *M* - log_10_ body mass; Abs. latitude (not analyzed further) – absolute latitude; *NPP avg* – average annual net primary productivity (t Carbon ha^−1^ y^−1^); *NPP max* – total NPP of most productive three months; *Prec avg* – avg monthly precipitation (mm); *Temp avg* – average annual temperature (°C); *Temp max* – average temperature of the warmest three months (°C); *PET avg* – average potential evapotranspiration (mm); *Aridity* – *Prec avg*/*PET avg*; *Temp range* – absolute difference between average January and July temperature (°C); *Prec range* – difference between average maximum and minimum monthly precipitation (mm); *Prec CV* – coefficient of variation of monthly precipitation across 30 years (%). All absolute values of r >0.38 are significant at p<0.001. Values≥0.5 are highlighted in bold (N = 97).

**Table 2:** Environment has strong effects on avian BMR, above and beyond migratory tendency.

	λ = 0			ML λ				
	AIC	b	p	AIC	b	p	λ	P (λ = 0)
*M*	−137.27	0.7288	**1.00E-9**	−151.18	0.7153	**1.00E-9**	0.82	1.92E-4
*Pass/non-P*		0.1233	**5.92E-4**		0.1437	**0.0935**		
*Migratory*		0.0611	**0.0124**		0.0355	0.1741		
*NPP avg*	−143.78	−0.0081	**0.0024**	−155.46	−0.0072	**0.0075**	0.81	6.31E-4
*NPP max*	−144.48	−0.0331	**0.0017**	−155.61	−0.0290	**0.0067**	0.80	8.52E-4
*Prec avg*	−141.51	−0.0018	**0.0078**	−154.92	−0.0017	**0.0099**	0.81	2.50E-4
*Temp avg*	−168.15	−0.0125	**1.38E-8**	−193.93	−0.0133	**1.00E-9**	0.93	3.83E-7
*Temp max*	−149.63	−0.0106	**0.0001**	−165.87	−0.0118	**3.40E-5**	0.89	5.56E-5
*PET*	−148.34	−0.0016	**0.0002**	−165.59	−0.0017	**3.91E-5**	0.87	3.29E-5
*Aridity*	−134.13	0.0242	0.7216	−148.82	0.0605	0.3508	0.83	1.27E-4
*Temp range*	−147.68	0.0039	**0.0003**	−171.99	0.0052	**1.28E-6**	0.91	1.38E-6
*Prec range*	−135.04	−0.0321	0.3223	−151.08	−0.0567	0.0782	0.84	6.06E-5
*Prec CV*	−134.00	−0.0001	0.9856	−149.28	−0.0003	0.3951	0.84	1.29E-4

BMR was first modeled as a function of body mass (*M*), passerine/non-passerine differences (*Pass/non-P*) and migratory tendency (*Migratory*). Controlling for these three variables, subsequently single environmental predictors were tested for their effect on BMR. The table shows the fitted parameter (*b*), the estimate of the AIC and the *P*-value testing whether the parameter is significantly different from zero. Parameters were estimated singly, and we conducted two analyses for each parameter: first one in which phylogeny was ignored (λ = 0), and then one in which we estimate Pagel's *λ*, and set it equal to its maximum likelihood value (ML λ). We tested whether the maximum likelihood estimate of *λ* was different from zero, i.e. whether the data show significant phylogenetic signal.

The observed environmental correlates may at least partially arise from the different environmental niches occupied by birds with different migratory strategies and associated differences in BMR. In our data, the mean absolute latitude occupied by migrants was 38.2°, whereas the corresponding values for non-migrants is 24.0°, and significantly lower. Similarly, the mean *Temp avg* for migrants is 15.21°C, and 19.14°C for non-migrants. Although the two groups do not differ in terms of *NPP*, *NPP max*, *Prec range* or *Prec CoV* they do in terms of the other environmental variables (Table 3): Temperature (maximum, average and range), *PET*, and *Aridity index* are all significantly associated with migratory tendency - this suggests that the negative effect temperature and associated variables have on avian BMR may at least partially be the result of the colder, more seasonal environments occupied by migrants with potentially higher BMR.

**Table 3:** Differences in environmental associations of migrants and non-migrants.

Variable	F_1,95_	p	λ	λ = 0, λ = 1
*NPP avg*	0.03	0.86	0.91	^*** .***^
*NPP max*	0.08	0.78	0.94	^*** .***^
*Prec avg*	1.98	0.16	0.96	^***. ***^
*Temp avg*	19.46	2.73E-5	0.76	^***. ***^
*Temp max*	9.93	2.20E-3	0.96	^***. ***^
*PET*	27.39	1.01E-6	0.95	^*** . ***^
*Aridity Index*	13.69	3.60E-4	0.99	^*** .***^
*Temp range*	10.51	1.65E-3	0.89	^*** .***^
*Prec range*	2.23	1.40E-1	0.99	^*** .**^
*Prec CoV*	2.15	1.46E-1	1.00	^***. ns^

We tested whether the average values of the environmental variables differed between migrant and non-migrant populations in the dataset, by fitting a linear model for each variable separately in which it was treated as the dependent variable and migratory tendency as a predictor. Shown is the F-ratio for the model, together with the P-value. For each model we estimated Pagel's *λ*, and set this equal to its maximum likelihood value. We tested whether this was different from zero and one (respectively as indicated by the superscripts) in order to determine whether significant phylogenetic signal existed (ns = not significant; ^*^ = p<0.05; ^**^ = p<0.01; ^***^ = p<0.0001). For other details see Table 2.

To investigate the generality of putative environmental correlates and their interactive effect with phylogeny we therefore repeated the single-predictor environment analyses separately for migrants and non-migrants. In migrants we found strong evidence for an influence of environmental variables, especially *Temp avg and PET*, *Aridity*, *Temp range* and *Prec range* (see Table 4). In all cases, accounting for phylogeny increased the estimated effect. However, the estimated values of *λ* were low (range 0 to 0.74) and in all cases were not significantly different from zero, indicating a low degree of phylogenetic dependence. In non-migrants the situation with respect to phylogeny is quite different: maximum likelihood values of *λ* are high and not significantly different from one (range 0.92 to 1; Table 4). There were somewhat fewer significant environmental correlates of BMR compared to migrants, with only *NPP max*, *Temp avg*, *Temp max*, and *Temp range* being significant in the models for non-migrants. In both groups heat related variables, specifically *Temp avg*, were the strongest predictors. While the statistical strength of *Temp avg* is much weaker than that of *M*, the variation of BMR along a temperature gradient is nevertheless considerable: a non-migrant bird at a location with on average 8°C annual temperature has basal energy fluxes that are 48% higher than a bird of the same size at a 28°C location (e.g. for a 10g passerine bird (95 c.i.): BMR (8°C) = 0.154 (±0.087) *W*, BMR (28°C) = 0.081 (±0.051) *W*, for *λ* = 0). We note that the models in Tables 4*a and 4b* include passerine/non-passerine differences, which are phylogenetic effects, and that the strength of this effect differs between migrants and non-migrants. However, the results we report are essentially the same when the analysis is repeated without this variable included (Table S1).

**Table 4:** Environmental correlates of BMR across migrants (a) and non-migrants (b) accounting for *M* and *Pass/non-P.*

	Migrants							
	λ = 0			ML λ				
	AIC	b	p	AIC	b	p	λ	P(λ = 0)
*M*	−109.95	0.737	**1.00E-8**	−109.95	0.737	**1.00E-8**	0.00	1.00
*Pass/non-P*		0.111	**0.0058**		0.111	**0.0058**		
*NPP avg*	−110.04	−0.0051	0.0895	−110.04	−0.0051	0.0895	0.00	1.00
*NPP max*	−109.38	−0.0187	0.1308	−109.38	−0.0187	0.1308	0.00	1.00
*Prec avg*	−110.81	−0.0023	0.0582	−110.81	−0.0023	0.0582	0.00	1.00
*Temp avg*	−125.70	−0.0104	**3.38E-5**	−126.33	−0.0125	**1.40E-6**	0.74	0.42
*Temp max*	−116.81	−0.0883	**0.0026**	−116.81	−0.0883	**0.0026**	0.00	1.00
*PET*	−123.88	−0.0019	**8.11E-5**	−125.32	−0.0026	**5.49E-6**	0.63	0.23
*Aridity*	−112.35	0.1616	**0.0255**	−112.35	0.1616	**0.0255**	0.00	1.00
*Temp range*	−110.20	0.0025	**0.0819**	−110.20	0.0025	**0.0819**	0.00	1.00
*Prec range*	−112.98	−0.1388	**0.0183**	−112.98	−0.1388	**0.0183**	0.00	1.00
*Prec CV*	−107.50	−0.0021	0.452	−107.50	−0.0021	0.452	0.00	1.00

	Non-migrants							
	λ = 0			ML λ				
	AIC	b	p	AIC	b	p	λ	P (λ = 0)
*M*	−40.31	0.707	**1E-16**	−49.76	0.680	**1E-16**	0.99	**0.0021**
*Pass/non-P*		0.127	**0.032**		0.138	0.278		
*NPP avg*	−43.58	−0.0107	**0.0168**	−52.27	−0.0089	**0.0219**	0.95	**0.0032**
*NPP max*	−44.63	−0.0441	**0.0098**	−53.21	−0.0370	**0.0137**	0.95	**0.0034**
*Prec avg*	−42.01	−0.0020	**0.0380**	−50.25	−0.0016	0.0559	0.92	**0.0041**
*Temp avg*	−53.90	−0.0140	**0.0001**	−62.11	−0.0120	**0.0002**	0.93	**0.0042**
*Temp max*	−43.88	−0.1245	**0.0143**	−54.36	−0.1220	**0.0084**	0.98	**0.0012**
*PET*	−41.25	−0.0013	0.0568	−50.62	−0.0011	0.0519	0.95	**0.0022**
*Aridity*	−38.79	−0.1562	0.2352	−47.16	−0.0853	0.4849	0.98	**0.0038**
*Temp range*	−45.59	0.0044	**0.0061**	−54.04	0.0041	**0.0090**	0.95	**0.0036**
*Prec range*	−37.47	−0.0218	0.6366	−49.11	−0.0634	0.1389	1.00	**0.0006**
*Prec CV*	−37.50	0.0002	0.6146	−46.88	0.0002	0.6640	0.99	**0.0022**

For other details see Table 2. For results.

We develop a final multi-predictor model of migrant and non-migrant BMR which confirms the importance of temperature: when fitted as a combined model the major environmental correlate of BMR is *Temp avg*, irrespective of migratory status (Table 5). The degree of phylogenetic dependence also varies between migrants and non-migrants. In the case of the former, the maximum likelihood value of *λ* is not different from zero, whereas in the latter case it is not significantly different from 1 (Table 5).

**Table 5:** Combined effects of select environmental variables on BMR in migrants and non-migrants.

Term	Non-Migrants			Migrants		
	*b*	*t*	*p*	*b*	*t*	*p*
*M*	0.6743	15.40	**1.64E-14**	0.7078	22.89	**1.00E-9**
*Pass/non-p.*	0.2149	2.10	**0.0419**	0.1602	2.55	**0.0142**
*Temp avg*	−0.0108	−3.34	**0.0019**	−0.0129	−5.48	**1.73E-6**
*Prec avg*	−0.0009	0.60	0.5514	−0.0061	−1.70	0.0949
*NPP avg*	−0.0073	−1.04	0.3041	0.0171	1.84	0.0720
	λ = 0.93 (λ = 0: p = 0.004, λ = 1: p = 0.52)			λ = 0.81 (λ = 0: p = 0.11, λ = 1: p = 2E-6)		
	*AIC = −57.40; N* = 45, *R* ^2^ = 0.88			*AIC = −123.67; N* = 52, *R* ^2^ = 0.93		

Models were fitted including all predictors simultaneously. Shown are the parameter estimates (*b*), and *t* and *p* values testing for difference of b from zero. For each model we accounted for phylogeny by including Pagel's *λ*, and setting this equal to its maximum likelihood value. We tested whether *λ* was different from zero and one in order to determine whether significant phylogenetic signal existed. For other details see Table 2.

## Discussion

### Migrants vs non-migrants

A considerable component of body mass-independent variation in avian BMR can statistically be explained by migratory tendency: the minimum normothermic maintenance energy requirements of migrants are significantly higher than those of non-migrants, at least when phylogeny is not accounted for. This has implications for comparative studies, since comparisons of the energetic traits of non-migrants with those of migrants may lead to misleading conclusions regarding physiological adaptation. The physiological divergence we have identified between migrant and non-migrant birds is consistent with observations that several species of migrant shorebirds have higher BMR than expected on the basis of body mass [30]–[32].

Why should BMR be higher in migrants than in non-migrant species? One possibility is that compared to species with more sedentary life histories the metabolic machinery for long-distance migration involves elevated maintenance costs. Avian energy intake rates as well as maximum thermogenic metabolic rates are positively correlated with BMR [46], [47]. A mechanistic link between elevated BMR and the capacity for sustained activity is supported by the observation that in Rock Doves (*Columba livia*), the metabolic intensity (cytochrome *c* oxidase activities) of pectoral muscles is higher in active birds than in sedentary individuals. The elevated BMR of migrant species may also in part reflect the timing of metabolic measurements and the influence of flight muscle hypertrophy preceding long-distance migratory flights in species such as Red Knots *Calidris canutus* [48].

**Figure 2:**
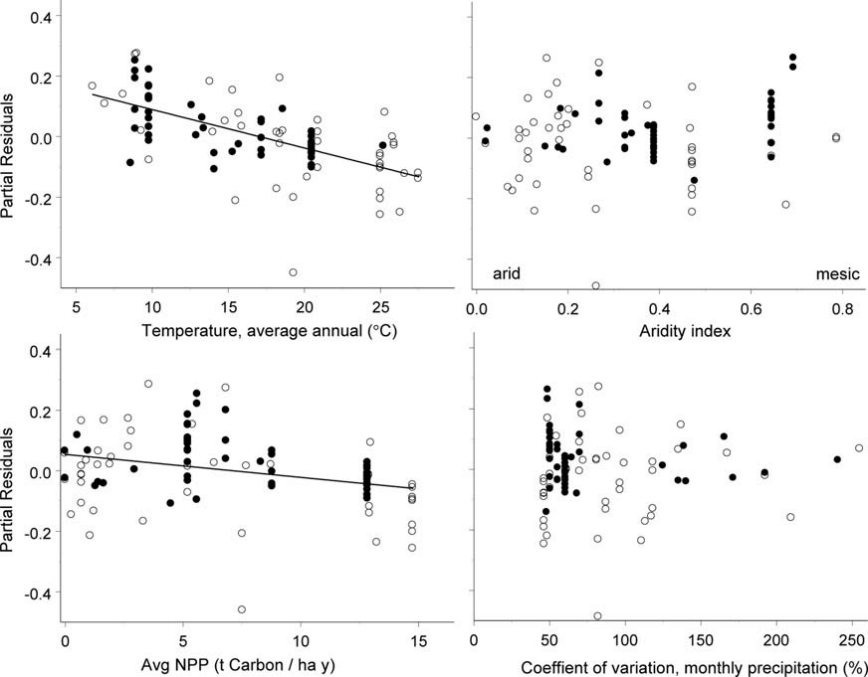
Partial residual plots of core environmental correlates of BMR for the combined dataset of both migrant (black circles) and non-migrant birds (open circles). Each panel shows the effect of a single predictor on BMR controlled for body size and Pass/non-p membership. Regression lines are those significant for combined non-migrant – migrant data (Table 2). Negative residual outlier (<−0.4 partial residual value) is the group-living Green Woodhoopoe (*Phoeniculus purpureus*).

A second set of possible explanations for the higher BMR of migrants compared to non-migrants concerns their uneven latitudinal distribution. During the breeding season migrants generally occur in environments that are colder (Figure 2) and where the short time window available for breeding may impose particularly high energy demands. According to the former view, the elevated BMR of migrants may simply reflect the correlation between BMR and *Temp avg*. This idea is not new; Kvist and Lindström [49] showed that the BMR of migrant shorebirds is highest on the Arctic breeding grounds before migration, and lowest while on tropical wintering grounds. Lindström and Klaassen [32] confirmed the generality of elevated BMR in shorebirds while in the Arctic, and hypothesized that the reduction of BMR exhibited by migrants as they move from high to tropical latitudes reflects changing requirements for thermoregulatory heat production. Many non-migrant species rapidly up- or down-regulate BMR in response to thermal acclimation [22], [23], [50], with BMR up-regulation associated with cold air temperatures and *vice versa*. Intra-individual variation in BMR often reflect changes in organ size [51], [52], but may also reflect changes in the metabolic intensity of specific tissues [53].

In our analysis, migrant data came from much higher latitudes and colder regions than data for non-migrants. In the combined non-migrant/migrant dataset several environmental variables exceed migratory tendency as predictor (Table 2): after accounting for either *Temp avg*, *PET* or *Temp range* there is no significant difference in BMR between migrants and non-migrants. In view of the considerable phenotypic flexibility in BMR exhibited by long-distance migrants [25], [32], [54], [55], and the consistent negative relationship between BMR and air temperature in laboratory acclimation studies [22], [23], [25], [50], it seems likely that the higher BMR of migrants we report here is determined in part by temperature effects. However, the BMR data currently available for long-distance migrants on their tropical wintering grounds are too few to rigorously test this hypothesis.

### Environmental correlates of avian BMR

Our analyses confirm the previously observed considerable body mass-independent variation in avian BMR that can be attributed to several environmental variables. For interpretation of specific environmental effects we focus on single-predictor environmental relationships as high collinearity of environmental variables (Table 1) hampers the interpretation of multi-predictor models. Average temperature (*Temp avg*) emerges as the most significant single environmental predictor of BMR, confirming the findings of White et al. (2007). BMR is significantly lower in warmer environments among all species included in our analysis, as well as within non-migrant and migrant subsets. These observations are consistent with Weathers' [14]'s finding that avian BMR increases with increasing latitude, Wiersma et al.'s [12] observation that tropical birds have lower BMRs than their temperate counterparts, as well as with the negative correlation between temperature and BMR in mammals [10]. A negative effect of *Temp avg* on avian BMR has also been demonstrated to explain intraspecific variation [16], [56]. Moreover, numerous studies of thermal acclimation or acclimatization have found that birds adjust BMR in response to changing thermoregulatory demands [21]–[23], [25], [50], [57], [58]. In studies involving thermal acclimation under laboratory conditions, BMR can be adjusted by more than 20% over time scales of several weeks [22], [25], [50].

Within and among migrants and non-migrants, BMR exhibited no correlation or a negative relationship with net primary productivity (which correlates positively with average temperature), a similar observation to that of White et al. (2007). This conflicts with the pattern among five species of *Peromyscus* mice under common-garden conditions [59]. Furthermore, the lack of a positive effect of habitat energy availability on avian BMR indicates that the apparent independence of individual energy demand and supply at broad scales found for field metabolic rate [20] is manifested at the level of BMR. An increase in maintenance metabolism with decreasing NPP could also potentially reflect greater mobility among desert species that have to cover larger areas to acquire sufficient resources to breed. Unpredictable fluctuations in food availability, driven by erratic rainfall, have traditionally been viewed as one of the major factors driving the evolution of low BMR in desert endotherms [60], and more recently have been invoked as a major determinant of zoogeographical patterns of mammalian BMR [8], [10] Whereas precipitation variability has been found to be strongly negatively associated with the BMR of small mammals [10], a recent study found the opposite for birds (White et al. 2007). Here we were not able to confirm any significant effect of precipitation variability on BMR. We interpret our results as evidence that the low BMR of desert bird species is determined primarily by temperature effects, rather than magnitude and variability in energy availability.

We emphasize, however, that our findings are correlational. BMR has long been viewed as a fixed, taxon-specific parameter, with adaptation inferred from interspecific variation remaining after scaling and/or phylogenetic patterns of descent have been accounted for. However, there is increasing evidence for considerable phenotypic flexibility in BMR, with substantial within-individual adjustments occurring over short time scales [61]. Thus, interspecific variation in BMR reflects in part the conditions to which birds are acclimatized or acclimated at the time of metabolic measurements, and not necessarily genotypic divergence.

### Disparate phylogenetic structure

Our results indicate a hitherto unappreciated complexity in the phylogenetic structure of the examined associations. Previous analyses of these types of data [40] and of BMR in birds in particular [13], [33] have assumed a common phylogenetic dependence across the whole data set analyzed. In this dataset migrants appear to have a much lower degree of phylogenetic dependence than non-migrants. This is consistent with the idea that in migrants post- and pre-migration changes in BMR within individuals cause intraspecific variation which then leads to BMR to appear phylogenetically more labile than in non-migrants (at least when measured in summer). Alternatively, it may also reflect a closer adaptation to more constant environmental conditions in migrants, but as we do not find much stronger environmental associations for them we believe this to be unlikely. These results suggest that future analyses may benefit from exploring more complex mosaic models of evolution and, ideally, datasets that include intraspecific genetic information.

### Conclusions

By analyzing BMR of migrants and non-migrants separately for a large number of species we have shed further light on an apparent pattern of physiological divergence among birds. The higher summer BMR of many migrant populations confirms that the observations of several authors working on migrant shorebirds [30]–[32] represent a general pattern. We find that the higher BMR of migrants may at least partly be due to the latitudinal distribution of their breeding grounds at higher latitudes and thus colder climates. While ambient temperature exerts a strongly negative effect on both migrant and non-migrant BMR, our analysis reveals that overall different environmental variables are correlated with the variation of BMR in these two groups. Our results confirm the need to consider and, ideally, to quantify environmental effects when addressing topics such as the body size dependence of metabolic rate or when developing models of population energy fluxes.

In conclusion it appears that broad-scale climatic gradients constraints, specifically those related to temperature, present a stronger constraint on avian BMR than migratory tendency. But different climatic associations and a much weaker phylogenetic signal point to different control of BMR in migrants that appears to be characterized by strong phenotypic flexibility. Further empirical work on winter vs. summer BMR in both migrants and non-migrants across environments will help to establish the full extent of phenotypic flexibility in each group and provide a fuller picture of its environmental determinants. Ultimately, such work may be extended to help understand the shapes and phylogenetic inertia of reaction norms across different migratory and other behavioral strategies. In our study we were able to only indirectly draw inference about the exact pathway that causes the statistically strong association between climate and phenotypic variation. But especially for migratory tendency the phylogenetically labile environmental control of BMR emphasizes the significance of phenotypic plasticity. Promising additional insights are likely to be gained from any study that was able to explicitly and simultaneously address environment - BMR association within individuals, within (and ideally across) populations and across species. Such studies, logistically challenging they may be, may be able to reconcile broad-scale comparative/eco-geographic perspectives that are concerned with the broad interspecific patterns with ecophysiological viewpoints that have helped appreciate the importance of small scale and intraspecific variation arising from phenotypic flexibility.

## Materials and Methods

### BMR and body mass data

We obtained BMR (Watts) and body mass (*M*, g) data for wild-caught populations of 135 species from McKechnie et al. [35]. We included estimates of BMR irrespective of the sample size from which they were generated, but tested for bias resulting from this approach (see below). For each datum, we consulted the original source for the location at which the experimental individuals were captured (online supporting material for McKechnie et al. 2006 at http://dx.doi.org/10.1098/rspb.2005.3415). In cases where the co-ordinates of the capture site were not reported, we obtained these from the Alexandria Library Digital Gazetteer (http://www.alexandria.ucsb.edu/gazetteer/). We classified each species as non-migrant (71 species) or migrant (64 species) using secondary literature and further separated migrants into those performing long-distance (inter-continental, 29 species) or short distance (intra-continental, 35 species) seasonal movements. Data included only birds captured on their breeding grounds (i.e., data from winter quarters are excluded). For the environmental analyses, we excluded 38 species from the dataset of McKechnie et al. [35] for which not all the environmental variables (see below) were available. This yielded a final data set of 97 species.

### Environmental predictor variables

For each BMR datum, we extracted the following environmental variables from the CRU CL2.0 data-set in 10-minute (0.167°) resolution and over the period 1961–1990 [37]: mean monthly temperature (*Temp avg*, °C), mean monthly temperature of hottest three months (*Temp max*, °C), mean monthly precipitation (*Prec avg*; mm/month), absolute difference between average January and July temperature (*Temp range*, °C), log_10_-transformed difference between average maximum and minimum monthly precipitation (*Prec range*, mm), and mean coefficient of variation of the monthly precipitation estimates across all 30 years (*Prec CoV*). We obtained estimates of net primary productivity (*NPP*, t Carbon ha^−1^ y^−1^) estimates for the period 1960–90 from the DOLY model in 0.5° resolution [38] and evaluate effects of average annual NPP (*NPP avg*, t Carbon ha^−1^ y^−1^) and average NPP of the most productive three months (*NPP max*, t Carbon ha^−1^ 3months^−1^). We also obtained average potential evapotranspiration (*PET avg*, mm) data from a 0.5° gridded global dataset [39]. PET is the amount of moisture which, if available, would be removed from a given land area by soil evaporation and plant transpiration. We used these data to calculate an aridity index (*Aridity Index*) as average monthly precipitation/average monthly PET, where moist areas have high aridity index and arid areas a low index. Several other variables measuring environmental conditions or their seasonality were evaluated, but excluded from the final analysis for their poor predictive ability or co-linearity with already included predictors.

### Model fitting and analysis – environmental effects on BMR

We initially analyzed the BMR data following McKechnie et al. [35], using a generalized least squares approach whereby covariance among species is accounted for using a phylogeny [40]–[42]. The strength of the phylogenetic signal in each model was assessed using the parameter λ which measures and controls for the degree of phylogenetic dependence in the model residuals [42], [43], estimated using a maximum-likelihood approach.

Our first set of analyses addresses the effects of single environmental predictors on avian BMR without distinguishing between migratory strategies. We fit general linear models (GLM) to the overall data set, using log_10_-transformed BMR as a continuous dependent variable, and successively add log_10_-transformed *M* (continuous) and passerine/non-passerine membership (*Pass/non-p.*, categorical). The passerine/non-passerine variable was added since there are significant differences in *M* between passerines and non-passerines, and omitting this variable leads to an under-estimation of the scaling exponents relating BMR to *M* [35], [44].

Environmental correlates on avian BMR may arise via the different geographic distributions and environmental associations of migratory vs. non-migratory species with different BMR. In the next set of analyses we therefore sought to examine how in this dataset environmental variables and thus environmental niches of birds and their migratory strategy interact with each other, and how this is affected by phylogeny. To do this we fitted linear models in which the environmental variables were individually treated as response variables, and migratory strategy used as a predictor. These analyses therefore test whether there are significant differences between migrants and non-migrants in terms of the broad-scale environments they occupy. We controlled for phylogeny in fitting these models and estimated the *λ* statistic, as described above, to determine whether there was phylogenetic signal in the patterns of environment occupancy.

### Model fitting and analysis – contrasting migrants and non-migrants

Next we repeated the previous analysis of single environmental correlates of BMR separately for migrants and non-migrants, with *M* and *Pass/non-p* as covariates and evaluated model fits using the Akaike Information Criterion [45]. Using the approach of analyzing single environmental predictors it was not clear whether the predictors that explain BMR variation are the same for migrants and non-migrants. This is because first, a large number of potential combinations of predictors exist many of which possess similar degrees of explanatory power; and second, the predictors are correlated with each other. Further complicating this analysis was the problem that the degree of phylogenetic dependence differs between the migrants and non-migrants. This makes it difficult to deal with both groups of species together in a single analysis. In the next stage of the analysis we therefore (i) reduced the set of predictors down to a core set of environmental variables measuring key aspects of the environment. Specifically we looked at the effects of average NPP, average temperature and average precipitation. (ii) We analyzed migrants and non-migrants separately to see whether the overall responses appeared different. (iii) We combined all variables into a single multiple regression analysis in order to examine the effects of all of these simultaneously and analyzed the effects using a sequential decomposition of variance. We introduced *M* and *Pass/non-p* as initial predictors. We then added *Temp_avg* as this was, according to the initial analysis, clearly the strongest environmental correlate of BMR. The other predictors were then added after this (note that the order in which these variables were added did not affect the results). We note that any broad-scale analysis of this sort is complicated by the overlap in information (collinearity) between alternative predictors such different environmental variables or environmental and behavioral-ecological determinants, and the cause - effect assumptions inherent in statistical modeling approaches.

### Model fitting and analysis – effects of BMR sample size

In the above analyses, we included BMR data irrespective of the number of individuals per population sampled. To verify that the inclusion of data measured in only one or two individuals did not bias the results, we repeated the environmental variable analyses described above after weighting the BMR and *M* data for each species by the number of individuals used. In no instance did the significance, direction or strength of an effect change. At most, partial coefficient values altered slightly after data were weighted.

## Supporting Information

Table S1 Environmental correlates of BMR across migrants and non-migrants, accounting only for body mass.
(0.07 MB DOC)
