# Supplementary material for: Environment, Migratory Tendency, Phylogeny and Basal Metabolic Rate in Birds
Source: PLoS One. 2008 Sep 23;3(9):e3261. doi: 10.1371/journal.pone.0003261 (PMC2533122; doi:10.1371/journal.pone.0003261)
Supplement: Table S1 — Environmental correlates of BMR across migrants and non-migrants, accounting only for body mass. (0.07 MB DOC) [file pone.0003261.s001.doc]

Table S1: Environmental correlates of BMR across migrants and non-migrants, accounting only for *M*. For other details see Table 4.

Migrants

|  | λ = 0 | | |  | ML λ | | | | |
| --- | --- | --- | --- | --- | --- | --- | --- | --- | --- |
|  | AIC | b | p |  | AIC | b | p | λ | P(λ = 0) |
| *M* | -104.86 | 0.685 | 1E-8 |  | -109.95 | 0.695 | 1E-8 | 0.47 | 0.3750 |
|  |
| *NPP avg* | -102.87 | -0.0039 | 0.228 |  | -103.06 | -0.0035 | 0.3338 | 0.41 | ns |
| *NPP max* | -102.42 | -0.0134 | 0.3091 |  | -102.61 | -0.0107 | 0.4773 | 0.41 | ns |
| *Prec avg* | -103.11 | -0.0017 | 0.1946 |  | -103.45 | -0.0016 | 0.2609 | 0.45 | ns |
| *Temp avg* | -113.22 | -0.0009 | **0.0091** |  | -123.42 | -0.00127 | **7.49E-07** | 0.87 | **0.0011** |
| *Temp max* | -106.04 | -0.0066 | **0.0358** |  | -109.73 | -0.0094 | **0.0048** | 0.69 | ns |
| *PET* | -110.51 | -0.0015 | **0.0035** |  | -119.62 | -0.0002 | **8.28E-6** | 0.83 | **0.0025** |
| *Aridity* | -106.60 | 0.1556 | **0.0456** |  | -107.42 | 2.04344 | **0.0254** | 0.52 | ns |
| *Temp range* | -106.85 | 0.0034 | **0.0231** |  | -109.25 | 0.0004 | **7.00E-3** | 0.66 | ns |
| *Prec range* | -106.89 | -0.1439 | **0.0225** |  | -106.89 | -0.1439 | **0.0225** | 0.00 | ns |
| *Prec CV* | -102.38 | -0.0003 | 0.3174 |  | -104.40 | -0.0004 | 0.1296 | 0.57 | ns |

Non-migrants

|  | λ = 0 | | |  | ML λ | | | | |
| --- | --- | --- | --- | --- | --- | --- | --- | --- | --- |
|  | AIC | b | p |  | AIC | b | p | λ | P (λ = 0) |
| *M* | -38.39 | 0.632 | 1E-16 |  | -51.54 | 0.666 | 1E-16 | 0.99 | **0.0021** |
|  |
| *NPP avg* | -37.74 | -0.0061 | 0.175 |  | -51.88 | -0.0075 | 0.0533 | 0.99 | **0.0001** |
| *NPP max* | -37.23 | -0.0259 | 0.1302 |  | -52.65 | -0.0315 | **0.0355** | 0.99 | **8.61E-5** |
| *Prec avg* | -36.08 | -0.0011 | 0.2679 |  | -50.04 | -0.0012 | 0.1412 | 0.97 | **0.0002** |
| *Temp avg* | -41.66 | -0.0094 | **0.0115** |  | -60.43 | -0.0011 | **0.0006** | 0.98 | **1.48E-5** |
| *Temp max* | -36.79 | -0.0069 | 0.17 |  | -54.45 | -0.0115 | **0.014** | 1.00 | **2.64E-5** |
| *PET* | -36.91 | -0.0010 | 0.1576 |  | -51.48 | -0.0001 | 0.0611 | 0.96 | **0.0001** |
| *Aridity* | -35.17 | -0.0844 | 0.5311 |  | -48.06 | -0.5475 | 0.7048 | 1.00 | **0.0003** |
| *Temp range* | -43.7 | 0.0048 | **0.0004** |  | -55.22 | 0.0004 | **0.0008** | 0.97 | **0.0007** |
| *Prec range* | -34.78 | -0.0082 | 0.8641 |  | -49.76 | -0.0558 | 0.1903 | 1.00 | **0.0001** |
| *Prec CV* | -34.84 | 0.0001 | 0.7689 |  | -48.03 | -0.0002 | 0.7277 | 1.00 | **0.0003** |
